# Periodontal Status and Disease Activity in Psoriatic Arthritis and Psoriasis: A Cross-Sectional Study

**DOI:** 10.5152/ArchRheumatol.2026.25089

**Published:** 2026-01-16

**Authors:** Bulent Akyuz, Hazal Durmus, Huseyin Baytımur, Oyku Tomay Aksoy, Gizem Basak, Ozlem Daltaban, Erkan Alpsoy, İlhan Sezer, Cahit Kacar

**Affiliations:** 1 Department of Rheumatology, Akdeniz University Hospital, Antalya, Türkiye; 2 Department of Periodontology, Akdeniz University Faculty of Dentistry, Antalya, Türkiye; 3 Department of Dermatology, Akdeniz University Hospital, Antalya, Türkiye; 4 Department of Rheumatology, İzmir Katip Celebi University Ataturk Education and Research Hospital, İzmir, Türkiye; 5 Department of Physical Medicine and Rehabilitation, Akdeniz University Hospital, Antalya, Türkiye

**Keywords:** Comorbidity, periodontitis, psoriasis, psoriatic arthritis

## Abstract

**Background/Aims:**

Psoriatic arthritis (PsA) and psoriasis (PS) are chronic inflammatory disorders characterized by significant systemic involvement and comorbidities. This study investigated the occurrence of periodontitis among PS and PsA patients and evaluated its associations with clinical and demographic factors.

**Materials and Methods:**

Comprehensive evaluations were performed, including the Maastricht Ankylosing Spondylitis Enthesitis Score (MASES) for all participants, along with PsA-specific assessments such as Psoriatic Arthritis and Composite Psoriatic Disease Activity Index (CPDAI). Dermatological and periodontal clinical parameters were recorded. The participants were organized into 2 groups: those with periodontitis and those without.

**Results:**

The study included 128 participants: 62 with PS and 66 with PsA. Although the prevalence of periodontitis was higher among PsA patients compared with those with PS (46.97% vs 30.65%), this difference did not reach statistical significance (*P* = .058). Elevated CPDAI (odds ratio [OR]: 1.38; *P* = .001) and MASES (OR: 1.39; *P* < .001) scores were significantly associated with the presence of periodontitis in PsA patients.

**Conclusion:**

In this study, patients with periodontitis demonstrated higher disease activity scores, underscoring the importance of incorporating periodontal assessment into the multidisciplinary management of psoriatic disease, particularly in patients with active disease.

**Main Points**

- Periodontitis was more frequent in psoriatic arthritis than in psoriasis, without statistical significance.
- Higher Maastricht Ankylosing Spondylitis Enthesitis Score and Composite Psoriatic Disease Activity Index scores were significantly associated with periodontitis in psoriatic arthritis patients.
- Male sex, smoking, and enthesitis severity emerged as independent risk factors for periodontitis.
- Periodontal assessment ought to be integrated into the multidisciplinary care of psoriatic patients exhibiting high disease activity.

## Introduction

Psoriasis (PS) is a chronic inflammatory disease characterized by inflammation of the dermis and epidermis, arising from the interaction of environmental factors and genetic predispositions.^1^ Its prevalence varies across countries, with global analyses reporting rates between 0.14% and 3%.^2^ Psoriatic arthritis (PsA) is a chronic systemic inflammatory disease marked by joint and enthesial inflammation, affecting 0.1%-1% of the population and approximately 20% of individuals with PS.^3^ Psoriasis and PsA are linked to a spectrum of comorbidities, including diabetes, metabolic syndrome, cardiovascular diseases, and inflammatory bowel diseases, emphasizing the central role of inflammation in these conditions.^4^^-^^7^

Periodontitis is a chronic, multifactorial inflammatory disorder primarily affecting gingival tissues, initiated at the gingival sulcus and subsequently progressing to a localized but severe inflammatory milieu. As the disease advances, it compromises the integrity of the adjacent alveolar bone, leading to reductions in mineralized tissue density, loss of periodontal attachment, and an increased risk of irreversible tooth loss.^8^ Microorganisms such as *Porphyromonas gingivalis* play a pivotal role in the disease pathogenesis.^9^ Periodontitis is not limited to oral health issues but is also associated with systemic conditions such as diabetes, rheumatoid arthritis, and cardiovascular diseases.^10^^-^^12^

Recent epidemiological and clinical studies have reported that patients with psoriasis and psoriatic arthritis are at increased risk of developing periodontitis.^13^^-^^21^ However, findings across studies remain heterogeneous, and statistical significance has not always been consistently demonstrated. The underlying mechanisms linking these conditions are thought to involve shared inflammatory pathways. Proinflammatory cytokines such as IL(interleukin)-17 and TNF(tumor necrosis factor)-α have been implicated in both psoriatic disease and periodontitis, and the IL-23/IL-17 axis is considered a central contributor.^22^^-^^28^ While this study did not directly investigate immunological mediators, previous evidence suggests that common inflammatory mechanisms may contribute to the co-occurrence of these disorders.

Importantly, although the prevalence of periodontitis in psoriatic patients has been described, the relationships between disease activity indices and periodontal involvement have been less systematically explored. This gap provided the rationale for the present study, which aimed to evaluate the prevalence of periodontitis and its associations with disease activity scores, treatment characteristics, and demographic factors in cohorts of patients with psoriasis and psoriatic arthritis.

## Materials and Methods

### Study Population

A cross-sectional study design was employed to capture the association between periodontal disease and psoriatic disease activity at a single point in time. While this approach does not establish causality, it allows for a comprehensive assessment of potential correlations in a well-defined cohort. This was a single-center study conducted at a tertiary-care hospital. The patients were recruited from the rheumatology and dermatology outpatient clinics of Akdeniz University Hospital, Antalya, Turkey. All eligible patients meeting the inclusion/exclusion criteria were invited to participate. Eligibility was restricted to participants aged 18 years and above. The participants included 66 patients who were diagnosed with PsA according to the Classification Criteria for Psoriatic Arthritis by a rheumatologist and 62 patients who were followed up for PS by a dermatologist. Exclusion criteria included pregnancy, diabetes, history of periodontal therapy within the last 6 months, and recent use of systemic antibiotics that could alter the periodontal microbiota in the past 3 months to minimize confounding factors. All participants involved underwent comprehensive examinations by a rheumatologist, dermatologist, and periodontist. The examiner conducting the periodontal assessments was blinded to each subject’s PS or PsA status. No participants were excluded due to missing data.

Demographic data, including age, gender, body mass index (BMI) (kg/m^2^), comorbidities, smoking status, education level, disease duration, and medication history, were recorded for each participant. Comorbidities such as hypothyroidism, hypertension, cardiovascular events, depression, and asthma were noted.

Rheumatological assessments included recording the Health Assessment Questionnaire, Visual Analog Scale for pain (VAS-pain; 0-10, 0 = no pain, 10 = unbearable pain), and Maastricht Ankylosing Spondylitis Enthesitis Score (MASES) scores for all participants. Additionally, PsA patients had their Ankylosing Spondylitis Quality of Life, Composite Psoriatic Disease Activity Index (CPDAI), and Disease Activity for Psoriatic Arthritis (DAPSA) scores calculated. Dermatological evaluations recorded Psoriasis Area and Severity Index (PASI), Nail Psoriasis Severity Index (NAPSI), Dermatology Life Quality Index (DLQI), and Body Surface Area scores for all participants. Patients were divided into 2 groups according to their periodontal condition: those with periodontitis and those without.

Patients were categorized into 2 groups based on treatment: those receiving conventional disease-modifying antirheumatic drugs (DMARDs) only (methotrexate, sulfasalazine, or leflunomide) and those receiving biologic therapies (TNF, IL-17, or IL-23 inhibitors), administered either as monotherapy or in combination with conventional DMARDs (cDMARDs).

### Laboratory Examinations

Blood plasma samples were obtained and analyzed for C-reactive protein (CRP) using a quantitative immunoturbidimetric method, whereas the erythrocyte sedimentation rate was determined via the Westergren method.^34^^,^^35^

### Periodontal Parameters

The diagnosis of periodontitis was based on established clinical and radiographic criteria. A single experienced periodontist, blinded to psoriatic disease status, performed all examinations using a calibrated Williams probe.^36^

The following parameters were recorded:^37^

- Plaque Index (PI): assessed at 4 surfaces per tooth (score 0-3).
- Bleeding on probing: proportion of sites with bleeding after probing.
- Probing depth (PD): distance from the gingival margin to the pocket base at 6 sites per tooth.
- Clinical attachment level (CAL): distance from the cementoenamel junction to the pocket base, reflecting cumulative attachment loss.

Periodontitis was defined as interdental CAL loss at ≥2 non-adjacent teeth, in line with accepted case definitions. Radiographs were used to confirm alveolar bone loss.^37^

### Statistical Analysis

All the statistical analyses were conducted utilizing SAS 9.4 software (SAS Institute Inc., Cary, NC, USA). Descriptive statistics were expressed as mean ± SD for variables with normal distribution and as median with interquartile range for those not following a normal distribution. The Shapiro–Wilk test was employed to evaluate the normality of continuous variables and skewness coefficients. Based on these assessments, variables that did not conform to normal distribution were analyzed using non-parametric methods. Differences between 2 independent groups were performed using Student’s *t*-test for continuous variables with normally distributed data and the Mann–Whitney *U*-test was applied when normality assumptions were not met. Categorical variables were compared using the chi-square test or Fisher’s exact test, depending on data distribution and sample size. From the logistic regression analysis, odds ratios (ORs) were calculated with a 95% CI. A *P*-value of less than .05 was considered indicative of statistical significance throughout analysis.

### Ethical Approval

The study received approval from the Ethics Committee of Akdeniz University Hospital (Decision No.: KAEK-663, Project no.: TSA-2023-6258) on November 09, 2022, and was conducted in compliance with the Declaration of Helsinki. Written informed consent was obtained from all participants before enrollment.

## Results

Among the 128 participants (76 females and 52 males; age: 20-73 years, mean ± SD: 47.5 ± 13.35 years), 62 had PS and 66 had PsA. An analysis of patient characteristics revealed no significant differences in gender distribution, smoking habits, or BMI between the groups. However, comorbidities were more common in PsA patients (*P* = .0209). Periodontitis was detected in 46.97% of PsA patients (31/66) and 30.65% of PS patients (19/62); the difference was not statistically significant (*P* = .058) (Table 1).

Patients with PsA exhibited notably elevated scores in HAQ and MASES scores (*P* < .0001). Periodontal evaluations, encompassing metrics such as the PI, BOP, PPD, CAL, and tooth count, revealed a significant reduction in tooth count among PsA patients (*P* = .0072), whereas other parameters did not demonstrate a significant difference (Table 1).

Of the 128 participants, 50 were diagnosed with periodontitis. Comparative analysis between patients with and without periodontitis revealed that MASES scores were markedly higher in those diagnosed with periodontitis (*P* = .0002). Among PsA patients, those with periodontitis had significantly higher MASES and CPDAI scores than those without periodontitis (Table 2).

In a multivariable logistic regression model initially including age, sex, education level, smoking status, disease duration, comorbidity, BMI, and MASES score, backward elimination retained male sex, smoking, and MASES as independent predictors of periodontitis. Male was significantly associated with an increased risk of periodontitis compared to females (OR = 4.79, 95% CI: 1.88-12.23, *P* = .001); in contrast, education level was not significant in the multivariate analysis (OR = 2.33; 95% CI: 0.95-5.72; *P* = .066). Smokers had a 2.70‐fold increased risk relative to non‐smokers (OR = 2.70, 95% CI: 1.14-6.41, *P* = .024), and each 1‐point increment in MASES was associated with a 34% increase in periodontitis odds (OR = 1.34 per point increase, 95% CI: 1.13-1.59, *P* = .001). In a subgroup analysis of PsA patients only, higher CPDAI scores were also independently associated with increased periodontitis risk (OR = 1.38 per point increase, 95% CI: 1.14-1.66, *P* = .001) (Table 3). Treatment type was included in the multivariate model but showed no significant association with periodontitis (*P* > .05).

When analyzed based on the type of treatment, patients receiving biological therapy demonstrated significantly lower PASI, DLQI, and NAPSI scores compared to those receiving DMARDs (*P* = .001, *P* = .0248, and *P* = .029, respectively). However, no significant differences were observed in periodontal parameters such as PD or CAL between treatment groups (*P* = .2933 and *P* = .2037, respectively) (Supplementary Table 1).

## Discussion

Earlier studies have established an association between periodontitis and psoriatic diseases;^13^^-^^17^ however, most have primarily addressed prevalence rather than the interplay between disease activity and periodontal involvement. Given the shared inflammatory pathways underlying these conditions, whether higher disease activity in PS and PsA contributes to an increased risk of periodontitis remains an important yet underexplored question. The present study aimed to evaluate the relationship between systemic inflammation, musculoskeletal disease burden, and periodontal health, thereby extending the current literature. Identifying such associations may support the integration of periodontal assessment into dermatologic and rheumatologic practice, ultimately contributing to multidisciplinary management strategies.

The Köbner phenomenon, characterized by lesion induction after trauma, suggests parallels with enthesitis, which may also arise from repetitive stress. Both processes share inflammatory pathways, notably IL-17 and TNF-α.^8^^,^^24^^,^^26^^,27^ Similarly, the periodontal ligament, which anchors the tooth to the alveolar bone, has been implicated in inflammatory mechanisms relevant to periodontitis,^38^ thereby suggesting a potential enthesis-related process. In the present cohort, MASES scores were higher among patients with periodontitis. Although prior studies have not directly examined the association between MASES and periodontitis, these findings support a possible link. By contrast, 1 study in ankylosing spondylitis reported no significant correlation,^39^ while another demonstrated strong associations between MASES and periodontal indices, including BOP and CAL.^40^ Collectively, these findings underscore the need for further exploration of common inflammatory pathways bridging psoriatic disease, enthesitis, and periodontal pathology.

Although the prevalence of periodontitis was numerically higher in PsA than in PS patients, the difference did not reach statistical significance (*P* = .0585). This should therefore be regarded as a non-significant trend, and larger studies are needed to clarify this association. Indeed, Egeberg et al^13^ demonstrated in a large cohort that PsA patients exhibited a higher incidence of periodontitis than both PS patients and healthy controls.

In periodontitis, elevated levels of proinflammatory cytokines such as IL-1, IL-2, IL-6, IL-17, and TNF-α have been identified in gingival crevicular fluid, with a notable reduction following periodontal treatment.^22^^-^^24^^,^^26^ Similar cytokine pathways are also central in the pathogenesis of PS and PsA.^25^^,^^27^^,^^30^ In this study, compared with patients receiving DMARD therapy, patients receiving biological therapy presented significantly lower disease activity scores and CRP levels. However, in contrast to previous studies that suggested a potential protective effect of anti-TNF agents on periodontal health in ankylosing spondylitis^41^^,^^42^ and PsA,^43^ these findings did not show a significant reduction in periodontitis frequency among biologics-treated patients. While biologics effectively suppress systemic inflammation, their role in modulating periodontal health remains uncertain. The lack of protective effect observed here may relate to differences in treatment duration, regimens, or patient characteristics. Future research should address the influence of immunosuppressive therapies, their mechanisms of action, and treatment timing on periodontal outcomes.

Using composite disease activity indices,^44^ it was observed that higher CPDAI scores were significantly associated with the presence of periodontitis. Although data directly linking PsA disease activity to periodontal health remain scarce, these findings are consistent with broader evidence showing associations between disease activity measures and systemic comorbidities. For instance, a longitudinal study of 189 PsA patients linked elevated DAPSA scores to long-term health risks,^45^ while severe periodontitis has been correlated with higher PASI scores in psoriasis.^17^ Taken together, these findings emphasize the potential clinical relevance of assessing comorbidities, including periodontal disease, in relation to psoriatic disease activity.

Previous studies have reported inconsistent results regarding tooth loss in psoriatic disease. A Norwegian study found a higher number of missing teeth in psoriasis patients compared with controls,^18^ whereas another study observed no difference among PS, PsA, and control groups.^17^ In the present cohort, PsA patients had fewer remaining teeth than those with PS, suggesting a greater overall oral health impact within the PsA group.

The strengths of this study include the use of multiple validated indices (CPDAI, DAPSA, and MASES) to comprehensively assess PsA disease activity and its correlation with periodontal health. Nonetheless, several limitations must be acknowledged. The relatively modest sample size, the inclusion of smoking as a confounder,^46^ and the absence of a healthy control group limit the generalizability of these findings. Additionally, the duration of biologic therapy was not recorded, which may influence periodontal outcomes and represents an additional limitation. Moreover, the cross-sectional design precludes causal inferences.

In conclusion, this cross-sectional study evaluated the prevalence of periodontitis in patients with PS and PsA and examined its associations with disease activity. Although PsA patients showed a higher prevalence of periodontitis, the difference did not reach statistical significance. Importantly, periodontitis was associated with higher disease activity indices, suggesting a link between systemic inflammation and periodontal involvement. These results highlight the potential value of incorporating periodontal assessment into the routine multidisciplinary care of patients with psoriasis and PsA. Future longitudinal studies with larger, well-characterized cohorts, including appropriate control groups, are warranted to clarify causal relationships and to determine whether targeted periodontal interventions may improve overall disease outcomes.

## Supplementary Materials

Supplementary Material

## Figures and Tables

**Table 1.** Comparison of Demographic, Clinical, and Periodontal Parameters in Psoriasis and Psoriatic Arthritis Patients

**Characteristics**	**PS (n = 62)**	**PSA (n = 66)**	***P***
Age (years) (mean ± SD, range)	48.5 ± 13.25, (20.0, 73.0)	46.7 ± 13.47, (21.0, 73.0)	.0088^a^
Diagnosis duration (mean ± SD, range)	16.1 ± 10.62, (2.0, 50.0)	12.84 ± 8.86, (1.0, 40.0)	.2094^a^
BMI (mean ± SD, range)	27.1 ± 4.18, (17.6, 35.9)	28.9 ± 4.69, (20.5, 42.2)	.4027^a^
Gender (F/M)	32/30	44/22	.0831^b^
Smoker, n (%)	26 (41.9)	25 (37.9)	.6394^b^
Comorbidity, n (%)	12 (19.4)	25 (37.9)	.0209^b^
Education level, n (%)			<.0001^b^
≤High school	24 (38.7)	48 (72.7)	
>High school	38 (61.3)	18 (27.3)	
Periodontal diagnosis, n (%)			.0585^b^
Periodontitis	19 (30.6)	31 (47.0)	
Without periodontitis	43 (69.4)	35 (53.0)	
Treatment type, n (%)			.0304^b^
cDMARDs	30 (45.5)	40 (64.5)	
bDMARD	36 (54.5)	22 (35.5)	
HAQ	0.1 ± 0.26, (0.0, 2.0)	0.3 ± 0.43, (0.0, 2.2)	<.0001^a^
MASES	1.1 ± 1.74, (0.0, 7.0)	3.4 ± 3.15, (0.0, 11.0)	<.0001^a^
ESR	12.5 ± 6.51, (2.0, 36.0)	15.5 ± 9.33, (2.0, 47.0)	.0928^a^
CRP	3.7 ± 4.89, (0.6, 26.0)	5.7 ± 8.25, (0.5, 42.9)	.1488^a^
Number of teeth	24.4 ± 4.66, (7.0, 28.0)	22.3 ± 5.68, (2.0, 28.0)	.0072^a^
PI	1.7 ± 0.66, (0.3, 3.0)	1.6 ± 0.67, (0.1, 3.0)	.2982^a^
BOP	0.6 ± 0.27, (0.1, 1.0)	0.6 ± 0.29, (0.1, 1.0)	.8367^a^
PD	2.4 ± 0.71, (1.6, 5.0)	2.6 ± 0.86, (1.2, 5.2)	.2177^a^
CAL	2.4 ± 0.72, (1.6, 5.0)	2.6 ± 0.88, (1.2, 5.2)	.2466^a^

bDMARD, biological disease-modifying antirheumatic drugs; BMI, body mass index; BOP, bleeding on probing; CAL, clinical attachment level; cDMARDs, conventional disease-modifying antirheumatic drugs; CRP, C-reactive protein; ESR, erythrocyte sedimentation rate; HAQ, Health Assessment Questionnaire; MASES, Maastricht Ankylosing Spondylitis Enthesitis Score; PD, pocket depth; PI, plaque index; PS, psoriasis.

^a^Mann–Whitney *U* *P*-value.

^b^Chi-square *P*-value.

**Table 2.** Comparison of Demographic, Clinical, and Periodontal Parameters According to Periodontal Diagnosis

**Periodontal Diagnosis**	**Periodontitis (n = 50)**	**Without Periodontitis (n = 78)**	*P*
Age (years)	50.0 ± 12.64, (22.0, 73.0)	46.0 ± 13.64, (20.0, 71.0)	.1337^a^
Gender (F/M)	22/28	54/24	.0046^b^
Education level, n (%)			.032^b^
≤High school	34 (68.0)	38 (47.7)	
>High school	16 (32.0)	40 (51.3)	
Smoker, n (%)	28 (56.0)	23 (29.5)	.0028^b^
Treatment type, n (%)			.6248^b^
cDMARDs	26 (52.0)	44 (56.4)	
bDMARD	24 (48.0)	34 (43.6)	
Diagnosis duration	13.4 ± 9.25, (1.0, 40.0)	14.6 ± 10.22, (1.0, 50.0)	.4405^a^
BMI	28.0 ± 4.35, (17.6, 41.7)	28.2 ± 4.67, (17.6, 42.2)	.9825^a^
HAQ	0.2 ± 0.39, (0.0, 2.2)	0.2 ± 0.38, (0.0, 2.0)	.2118^a^
VAS-pain	2.8 ± 2.48, (0.0, 9.0)	2.0 ± 2.01, (0.0, 7.0)	.0763^a^
MASES	3.4 ± 3.06, (0.0, 11.0)	1.6 ± 2.37, (0.0, 10.0)	.0002^a^
ESR	13.9 ± 9.21, (2.0, 47.0)	14.1 ± 7.54, (2.0, 36.0)	.5589^a^
CRP	5.1 ± 7.11, (0.5, 40.8)	4.5 ± 6.77, (0.6, 42.9)	.7012^a^
Number of teeth	23.1 ± 5.59, (2.0, 28.0)	23.5 ± 5.13, (6.0, 28.0)	.6687^a^
PI	1.9 ± 0.67, (0.5, 3.0)	1.5 ± 0.61, (0.1, 3.0)	.0005^a^
BOP	0.7 ± 0.26, (0.3, 1.0)	0.5 ± 0.27, (0.1, 1.0)	.0003^a^
PD	3.2 ± 0.77, (1.9, 5.2)	2.0 ± 0.30, (1.2, 2.8)	<.0001^a^
CAL	3.2 ± 0.80, (1.9, 5.2)	2.0 ± 0.31, (1.2, 2.8)	<.0001^a^
PASI	2.9 ± 4.52, (0.0, 19.2)	2.2 ± 3.74, (0.0, 19.5)	.5443^a^
DLQI	5.3 ± 6.25, (0.0, 20.0)	4.9 ± 5.29, (0.0, 19.0)	.9777^a^
NAPSI	2.5 ± 3.69, (0.0, 16.0)	1.4 ± 2.66, (0.0, 14.0)	.0923^a^
BSA	2.7 ± 3.96, (0.0, 15.0)	2.2 ± 3.40, (0.0, 15.0)	.3875^a^
**Periodontal Diagnosis (PSA)**	**Periodontitis (n = 31)**	**Without Periodontitis (n = 35)**	
Treatment type, n (%)			.5890^b^
cDMARDs	13 (41.9)	17 (48.6)	
bDMARD	18 (58.1)	18 (51.4)	
MASES	4.4 ± 3.02, (0.0, 11.0)	2.5 ± 3.00, (0.0, 10.0)	.0057^a^
BASDAI	2.7 ± 2.12, (0.0, 8.0)	2.8 ± 1.85, (0.0, 7.4)	.6616^a^
ASQoL	3.6 ± 3.59, (0.0, 14.0)	3.5 ± 3.82, (0.0, 13.0)	.7802^a^
DAPSA	15.2 ± 11.71, (2.0, 54.0)	11.6 ± 9.86, (2.0, 49.0)	.1238^a^
CPDAI	4.8 ± 2.71, (0.0, 12.0)	2.8 ± 2.07, (0.0, 8.0)	.0016^a^
PASI	2.1 ± 3.82, (0.0, 17.0)	1.4 ± 2.31, (0.0, 10.8)	.8782^a^
DLQI	4.1 ± 5.85, (0.0, 19.0)	4.1 ± 5.01, (0.0, 18.0)	.9126^a^
NAPSI	2.4 ± 4.09, (0.0, 16.0)	1.5 ± 3.01, (0.0, 14.0)	.6394^a^

ASQoL, Ankylosing Spondylitis Quality of Life; bDMARD, biological disease-modifying antirheumatic drugs; BMI, body mass index; BOP, bleeding on probing; BSA, body surface area; CAL, clinical attachment level; cDMARDs, conventional disease-modifying antirheumatic drugs; CPDAI, Composite Psoriatic Disease Activity Index; CRP, C-reactive protein; DAPSA, Disease activity for psoriatic arthritis; DLQI, Dermatology Life Quality Index; ESR, erythrocyte sedimentation rate; HAQ, Health Assessment Questionnaire; MASES, Maastricht Ankylosing Spondylitis Enthesitis Score; NAPSI, Nail Psoriasis Severity Index; PASI, Psoriasis Area and Severity Index; PD, pocket depth; PI, plaque index; PS, psoriasis; VAS, Visual Analog Scale.

^a^Mann–Whitney *U* *P*-value.

^b^Chi-square *P*-value.

**Table 3.** Multivariate Logistic Regression for Periodontal Diagnosis

**Parameters**	**Odds Ratios**	**95% CI**	***P***
Gender (Male vs Female)^†^	4.79	1.88-12.23	.001
Education (≤High school vs. >High)^‡^	2.33	0.95-5.72	.066
Smoker (Yes vs. No)^¶^	2.70	1.14-6.41	.024
MASES (per 1‐point increase)	1.34	1.13-1.59	.001
CPDAI* (per 1‐point increase)	1.38	1.14-1.66	.001

CPDAI, Composite Psoriatic Disease Activity Index; MASES, Maastricht Ankylosing Spondylitis Enthesitis Score.

^†^Reference = Female.

^‡^Reference = Education > High school.

^¶^Reference = Non‐smoker.

*Analyzsed in PsA patients only.

## Data Availability

The data that support the findings of this study are available on request from the corresponding author.
