# Supplementary material for: Periodontal Status and Disease Activity in Psoriatic Arthritis and Psoriasis: A Cross-Sectional Study
Source: Arch Rheumatol. 2026 Jan 16;41(1):14–21. doi: 10.5152/ArchRheumatol.2026.25089 (PMC12869726; doi:10.5152/ArchRheumatol.2026.25089)
Supplement: Supplementary Material [file supplementary_material.pdf]

Supplementary Table 1. Associations of Treatment Type with Disease Activity and Periodontal Parameters in PS and PSA Patients

**Medication**	**DMARDs (n:70)**	**Biological therapy (n:58)**	***P***
Age	46.7±13.47, (21.0, 73.0)	48.5±13.25, (20.0, 73.0)	0.5134[a]
Diagnosis duration	12.84±8.86, (1.0, 40.0)	161±10.62, (2.0, 50.0)	0.0355[a]
ESR	15.0±7.44, (3.0, 36.0)	12.8±8.93, (2.0, 47.0)	0.0351[a]
CRP	5.8±7.09, (0.5, 40.8)	3.5±6.47, (0.6, 42.9)	0.0007[a]
Number of teeth	23.9±4.54, (7.0, 28.0)	22.6±6.08, (2.0, 28.0)	0.3802[a]
PI	1.7±0.65, (0.6, 3.0)	1.6±0.69, (0.1, 3.0)	0.5784[a]
BOP	0.6±0.28, (0.1, 1.0)	0.6±0.29, (0.1, 1.0)	0.8700[a]
PD	2.6±0.84, (1.7, 5.0)	2.4±0.73, (1.2, 5.2)	0.2933[a]
CAL	2.6±0.84, (1.7, 5.0)	2.4±0.76, (1.2, 5.2)	0.2037[a]
PASI	3.2±4.50, (0.0, 19.5)	1.5±3.25± (0.0, 19.2)	0.0010[a]
DLQI	6.0±6.03, (0.0, 20.0)	3.8±4.96, (0.0, 19.0)	0.0248[a]
NAPSI	2.2±3.21, (0.0, 16.0)	1.4±3.03, (0.0, 14.0)	0.0290[a]
BSA	3.4±4.26, (0.0, 15.0)	1.2±2.19, (0.0, 10.0)	0.0004[a]
**Medication (PSA)**	**DMARDs (n:30)**	**Biological therapy (n:36)**	
BASDAI	2.6±1.65, (0.0, 6.0)	2.9±2.21, (0.0, 8.0)	0.9332[a]
ASQoL	3.6±3.56, (0.0, 13.0)	3.5±3.84, (0.0, 14.0)	0.5070[a]
DAPSA	14.2±11.12, (2.0, 54.0)	12.6±10.69, (2.0, 49.0)	0.3766[a]
CPDAI	3.6±2.33, (0.0, 8.0)	3.9±2.80, (0.0, 12.0)	0.7751[a]

ASQoL - Ankylosing Spondylitis Quality of Life, DAPSA - Disease Activity for Psoriatic Arthritis, CPDAI - Composite Psoriatic Disease Activity Index, PI - Plaque Index, PD - probing Pocket Depth, CAL - Clinical Attachment Level, BOP - Bleeding on Probing, CRP - C-reactive protein, ESR – erythrocyte sedimentation rate, PASI - Psoriasis Area and Severity Index, DLQI - Dermatology Life Quality Index, NAPSI - Nail Psoriasis Severity Index, BSA - Body Surface Area, DMARDs - disease-modifying antirheumatic drugs.

[a]Mann–Whitney U *P*-value.
